# Identification of Promising Genotypes Through Systematic Evaluation for Arsenic Tolerance and Exclusion in Rice (*Oryza sativa* L.)

**DOI:** 10.3389/fpls.2021.753063

**Published:** 2021-10-29

**Authors:** Varunseelan Murugaiyan, Jauhar Ali, Michael Frei, Frederike Zeibig, Ambika Pandey, Andriele Wairich, Lin-Bo Wu, Jayaseelan Murugaiyan, Zhikang Li

**Affiliations:** ^1^Rice Breeding Platform, International Rice Research Institute (IRRI), Los Baños, Philippines; ^2^Institute of Crop Sciences and Resource Conservation (INRES), University of Bonn, Bonn, Germany; ^3^Department of Agronomy and Crop Physiology, Institute for Agronomy and Plant Breeding, Justus Liebig University Giessen, Giessen, Germany; ^4^Programa de Pós-Graduação em Biologia Celular e Molecular, Centro de Biotecnologia, Universidade Federal Do Rio Grande Do Sul, Porto Alegre, Brazil; ^5^Department of Biological Sciences, SRM University-AP, Amaravati, India; ^6^National Key Facility for Crop Gene Resources and Genetic Improvement, Institute of Crop Science, Chinese Academy of Agricultural Sciences (CAAS), Beijing, China

**Keywords:** arsenic toxicity, arsenic tolerance, arsenic exclusion, contaminated soil, seed germination, grain arsenic

## Abstract

Rice remains a major staple food source for the rapidly growing world population. However, regular occurrences of carcinogenic arsenic (As) minerals in waterlogged paddy topsoil pose a great threat to rice production and consumers across the globe. Although As contamination in rice has been well recognized over the past two decades, no suitable rice germplasm had been identified to exploit in adaptive breeding programs. Therefore, this current study identified suitable rice germplasm for As tolerance and exclusion based on a variety of traits and investigated the interlinkages of favorable traits during different growth stages. Fifty-three different genotypes were systematically evaluated for As tolerance and accumulation. A germination screening assay was carried out to identify the ability of individual germplasm to germinate under varying As stress. Seedling-stage screening was conducted in hydroponics under varying As stress to identify tolerant and excluder genotypes, and a field experiment was carried out to identify genotypes accumulating less As in grain. Irrespective of the rice genotypes, plant health declined significantly with increasing As in the treatment. However, genotype-dependent variation in germination, tolerance, and As accumulation was observed among the genotypes. Some genotypes (WTR1-BRRI dhan69, NPT-IR68552-55-3-2, OM997, and GSR IR1-5-Y4-S1-Y1) showed high tolerance by excluding As in the shoot system. Arsenic content in grain ranged from 0.12 mg kg^−1^ in Huang-Hua-Zhan (*indica*) from China to 0.48 mg kg^−1^ in IRAT 109 (*japonica*) from Brazil. This current study provides novel insights into the performance of rice genotypes under varying As stress during different growth stages for further use in ongoing breeding programs for the development of As-excluding rice varieties for As-polluted environments.

## Introduction

The global population continues to grow steadily and could reach 9.7 billion by 2050, which also demands a 50% increase in crop output over current levels (Li et al., 2014; Ali et al., 2021). Rice (*Oryza sativa* L.) is one of the most vital staple grains of the world, 90% of which is produced and consumed in Asian countries, and it influences the livelihoods of several billion people (Maclean et al., 2003; Wang et al., 2018). Conventionally, rice is grown in waterlogged paddy soils, which form a source of both beneficial (iron and zinc) and detrimental (arsenic, cadmium, and lead) mineral elements for the food chain (Murugaiyan et al., 2019). The presence of naturally occurring carcinogenic arsenic (As) minerals in waterlogged paddy soils poses a substantial challenge to rice production and threatens human and livestock health (Norton et al., 2012; Murugaiyan et al., 2019). Among Asian countries, Bangladesh and West Bengal regions of India are in most urgent need of As-excluding rice varieties to address the naturally occurring As contamination problem in their irrigation waters and soil (Panaullah et al., 2009). Rice is the staple diet for 156 million people in Bangladesh and the annual per capita rice consumption exceeds 152 kg (Biswas and Naher, 2019). In these major rice-growing belts, up to 40 million people are directly exposed to this class I carcinogen (Hossain, 2006; Rahaman et al., 2013). Elevated exposure to As from air, water, soil, and food has been linked to adverse health effects such as cancer and cardiovascular disease, and it inhibits the mental development of children (Mukherjee and Bhattacharya, 2001). Moreover, intake of inorganic As over a continuous period will cause the chronic As-poisoning illness called arsenicosis (Guha Mazumder, 2008). High concentrations of As have been found in paddy soils irrigated with As-contaminated groundwater. Apart from groundwater sources, perennial rivers originating from the greater Himalayas also carry As from their rock sediments into the densely inhabited major rice-growing floodplains of South and Southeast Asia (Polizzotto et al., 2013). Arsenic contamination in rice is a problem that is not limited to Bangladesh and India alone but is widespread across the major river plains of South and Southeast Asia (Polizzotto et al., 2008). In the Ganges-Brahmaputra deltas of India and Bangladesh, the average concentration of As in agricultural topsoil has been reported at 1.5–19 mg kg^−1^ and it can reach 83 mg kg^−1^ in highly contaminated regions (Saha and Rahman, 2020). In many of these areas, the groundwater As content is 100 times higher than the WHO guideline of 10 ppb for drinking water (George et al., 2014). However, no such limits were proposed for irrigation water. This leaves no alternative but to develop As-excluding rice varieties, keeping As content in both grain and straw within safe limits for human and livestock consumption.

The occurrence of As in the topsoil and irrigation water prompts various negative responses in rice plants, and these problems are often combined with other soil-related problems associated with rice cultivation (Ali et al., 2020). The presence of inorganic As in the paddy topsoil triggers various undesirable responses such as poor germination, stunted growth, discoloration, decreased photosynthetic activity, poor root establishment, and lower biomass, and the sterility-related physiological disorder straighthead disease was also associated with As toxicity (Zhao et al., 2013; Duncan et al., 2017). The toxic effects of As in rice depend on its chemical form, with organic As species being less toxic and bioavailable than the inorganic As form. Rice plants are quite efficient in taking up and translocating inorganic As species into their system (Murugaiyan et al., 2019). In natural waters and paddy topsoil, inorganic As exists in two main species forms, namely, more toxic trivalent arsenite As^(III)^ and less toxic pentavalent arsenate As^(V)^ (Abedin et al., 2002b). In irrigated lowland submerged paddy soils (anaerobic conditions), As predominantly exists as oxyanions of reduced arsenite As^(III)^ and in rainfed upland paddy soils (aerobic conditions), oxidized arsenate As^(V)^ dominates (Suriyagoda et al., 2018; Murugaiyan et al., 2021). Since rice plants do not possess any natural As transporters or have lost them during the domestication process, their uptake and translocation depend on exploiting closely resembling nutrients (Sahoo and Mukherjee, 2014). Arsenate As^(V)^ is physicochemically analogous to the essential nutrient phosphorus (P), and arsenite As^(III)^ uses the active uptake linked to aquaglyceroporin mediate acquisition pathways to gain access into the plant system (Ma et al., 2008; Zhang et al., 2008). Arsenate As^(V)^ can substitute for inorganic phosphate in a variety of biochemical processes that affect key metabolism in the cell. In contrast, As^(III)^ having high affinity toward sulfhydryl-containing enzymes interferes with key enzymes in a deleterious way (Murugaiyan et al., 2021).

More than 56% of the irrigated area of crop production of the world is found in Asia, of which 40–46% is used for irrigated lowland rice production and receives about 40% of available irrigation water of the world (Jewel et al., 2019). Since 35–55% of rice is produced in irrigated lowland, inorganic arsenite As^(III)^ constitutes the major As species loaded into rice plants (Murugaiyan et al., 2019). However, in recent times, rice-growing Asian nations have been adopting cost-efficient sustainable technologies for rice production such as alternate wetting and drying (AWD), and direct-seeded rice (DSR) (Mishra et al., 2017; Sander et al., 2017). Arsenic dynamics in these conditions are poorly understood and no suitable rice varieties for As-contaminated regions have been identified. For DSR production systems, the presence of inorganic As in the soil may significantly affect the most sensitive seed germination and establishment processes (Tang et al., 2020). When rice is grown in the monsoon wet season, dry-seeded rice is likely to be flooded (anaerobic conditions) most of the time. Thus, inorganic arsenite As^(III)^ species dominate (Carey et al., 2010). When rice is grown in the dry season with only a little irrigation or no irrigation (aerobic conditions), inorganic arsenate As^(V)^ species dominate (Zhao et al., 2010). Also, in recent times, advanced water management practices such as AWD were widely used in rice production (Sander et al., 2017). During AWD use, As tends to switch rapidly between the species As^(III)^ ⇌ As^(V)^. Arsenate As^(V)^ dominates during drying and arsenite As^(III)^ dominates during wetting (Norton et al., 2019; Yang et al., 2019). This switch between species is likely to be influenced by water availability, As bioavailability in soil, and the presence of microbial activity. Although As interaction with rice has been well recognized over the past two decades, its genotypic variations, the most significant tolerant varieties, screening strategies, and accumulation are poorly understood (Murugaiyan et al., 2021). So far, no donor germplasm associated with As exclusion has been identified or used in breeding programs for the development of As-excluding rice varieties. Many farmers in acutely As-contaminated areas are extremely poor and cannot afford costly mitigation strategies and alternative food (Murugaiyan et al., 2019).

In this current study, 53 varieties from the core breeding panel of the IRRI-GSR (International Rice Research Institute–Green Super Rice) program were systematically evaluated under varying As stress aiming at understanding the genetic variation in germplasm for germination capacity, As tolerance, shoot exclusion, and low grain accumulation. We combined three different screening strategies to recognize appropriate genotypes for As tolerance and exclusion during different growth stages. We hypothesized that the selected rice genotypes would show a diverse range of morphological and As accumulation trait variability for As-induced toxicity stress. The main objectives of this study were to (a) identify the germination ability of 53 genotypes facing varying As stress and cluster them into different As stress-tolerance groups, (b) explore the genetic potential of individual genotypes for As tolerance and exclusion at the early growth stage of rice and cluster them into different As stress-tolerance groups by shoot exclusion and inclusion groups, and (c) identify the most significant genotypes that accumulate less As in grain and categorize them into different As grain-accumulating groups. By combining these three different screening strategies, we explored the interactions of As tolerance and exclusion traits during different growth stages.

## Materials and Methods

### Plant Materials

Fifty-three genotypes involving seven IRRI-GSR advanced fixed lines and 46 different rice cultivars from the core breeding collection of the IRRI-GSR breeding program (Table 1) were systematically evaluated for As tolerance and exclusion. These 46 rice cultivars originated from the major rice-growing regions of China, India, the Philippines, Vietnam, Bangladesh, Sri Lanka, Pakistan, the United States, Brazil, and Iran where As contamination has been widely reported in waterlogged paddy topsoil (Bastías and Beldarrain, 2016; Bakhat et al., 2017). Seven IRRI-GSR genotypes were developed to resist various biotic and abiotic stresses across the rice-growing ecosystem. In addition, these diverse cultivars include the parents of several permanent mapping populations developed in the IRRI-GSR program for yield improvement under different unfavorable rice-growing environments (Ali et al., 2018).

**Table 1:** Description of plant cultivars used for arsenic tolerance screening.

**Cultivar name**	**Subspecies**	**Country of origin**	**Cultivar name**	**Subspecies**	**Country of origin**
GSR IR1-8-S6-S3-Y2 (GSR 8)	*Indica* (IRRI-GSR)	Philippines	Basmati	*Indica*	India
GSR IR1-12-Y4-D1-Y2	*Indica* (IRRI-GSR)	Philippines	Jhona 349	*Aus*	India
GSR IR1-12-D10-S1-D1 (GSR 12)	*Indica* (IRRI-GSR)	Philippines	Binam	*Aromatic*	Iran
GSR IR1-17-Y16-Y3-S1	*Indica* (IRRI-GSR)	Philippines	Basmati 385	*Aromatic*	Pakistan
GSR IR1-5-Y4-S1-Y1	*Indica* (IRRI-GSR)	Philippines	M401	*Temperate japonica*	United States
GSR IR1-5-Y7-Y2-SU1	*Indica* (IRRI-GSR)	Philippines	X21	*Indica*	Vietnam
GSR IR1-4-S5-L1-L1	*Indica* (IRRI-GSR)	Philippines	IRAT 109	*Tropical japonica*	Brazil
OM1706	*Indica*	Vietnam	Gayabyeo	*Indica*	China
IR64	*Indica*	Philippines	Shwe-Thwe-yin-Hye	*Indica*	China
OM1723	*Indica*	Vietnam	NAN29-2	*Temperate japonica*	India
IRAT352	*Indica*	Philippines	FR13-A	*Aus*	India
Phalguna	*Indica*	India	Jiangxi-Si-Miao	*Indica*	China
Zhong413	*Indica*	China	TKM9	*Indica*	India
Teqing	*Indica*	China	PSBRc82	*Indica*	Philippines
BR24	*Indica*	Bangladesh	NPT (IR68552-55-3-2)	*Tropical japonica*	Philippines
PSBRc66	*Indica*	Philippines	Huang-Hua-Zhan (HHZ)	*Indica*	China
CDR22 (IR50/Minghui 63)	*Indica*	China	Hua 565	*Indica*	China
WTR 1 (BRRI dhan 69)	*Indica*	China	Bg304	*Indica*	Sri Lanka
Feng-Ai-Zhan	*Indica*	China	Kalimekri 77/5 (6527)	*Indica*	China
Qi-Si-Ying-Zhan	*Indica*	China	BR11	*Indica*	Bangladesh
Xing-Ying-Zhan	*Indica*	China	IR66	*Indica*	Philippines
Te-Huang-Zhan	*Indica*	China	Thadokkham 1 (TDK1)	*Indica*	Lao PDR
Y-134	*Indica*	China	Feng Fu Zhan (FFZ)	*Indica*	China
Haoannong	*Tropical japonica*	China	TME80518	*Indica*	China
Cheng-Hui 448	*Indica*	China	Zhongzu 14	*Indica*	China
Bg300	*Indica*	Sri Lanka	SACG-4	*Indica*	China
OM997	*Indica*	Vietnam			

### Germination Screening Assay for Arsenic Tolerance

The seed germination screening assay was carried out in the plant growth chamber facility at IRRI for evaluating the germination tolerance of genotypes under varying As treatments. Five concentrations of inorganic As treatments, 0 ppm (control), 5 ppm (low), 10 ppm (medium), 15 ppm (high), and 20 ppm (very high), were supplied in the form of sodium arsenate (AsNaO_2_, Sigma Aldrich, Singapore) to evaluate the germination ability of seeds. All the purified seeds of the rice genotypes were oven-dried for 5 days at 60°C to break any residual seed dormancy and were surface-sterilized with 1% sodium hypochlorite (NaOCl) for 1 min, followed by rinsing with deionized water several times. Germination was evaluated on a moist filter paper (Whatman No. 1) bed dampened with 10 ml of the respective As treatment placed on each of the 15-cm-diameter Petri dishes. Fifty sterilized healthy seeds per genotype were laid on each Petri dish and incubated at room temperature (28–32°C) for germination. Each treatment was replicated three times and the seeds were allowed to germinate for 10 complete days. During this period, the Petri dishes were moistened with the respective solutions of As when required. After 10 days of incubation, the number of seeds germinated was recorded by the emergence of the radicle and coleoptile for the respective treatments and controls. Based on the germination percentage under different As treatments, the genotypes were placed into four different categories: highly tolerant (>80% germination), moderately tolerant (>50% germination), moderately susceptible (<50% germination), and highly susceptible (<20% germination). The tolerance percentage of the genotypes was calculated with the following equation:

$$\begin{array}{r} Arsenic\;tolerance\;index\;\left(germination\;\%\right)\\ =\frac{Germination\;observed\;in\;each\;arsenic\;treatment}{Germination\;observed\;in\;the\;control}\times \;100 \end{array}$$

### Seedling-Stage Screening for Arsenic Tolerance

Seedling-stage As stress screening experiments were conducted in a hydroponic system in the controlled phytotron glasshouse facility at IRRI. Optimum rice-growing conditions were maintained throughout the experiment: 29/21°C (day/night) temperature, 70% relative humidity, and natural light. Seeds of the 53 genotypes were oven-dried for 5 days at 60°C to break any residual seed dormancy and incubated at 30°C for 48 h for pre-germination. One seedling per genotype was transferred per well with 1 cm diameter on a Styrofoam seedling tray with the size of 28 × 32 × 1.25 cm having 100 wells (10 × 10) with a nylon net bottom fixed in a dark plastic tray containing 8 L of full-strength Yoshida nutrient solution (Wu et al., 2017). On the seventh day, 5 ppm As, 10 ppm As, and 15 ppm As were supplied in the form of sodium-meta-arsenite (AsNaO_2_, Sigma-Aldrich, MO, USA). The control treatment without As in the nutrient solution was maintained throughout the experiment. The plants were grown under As-toxic conditions for 18 days, with the pH adjusted to 5.4 every other day and nutrient solutions were completely renewed every seventh day. The experiment was laid out as a complete randomized design with three independent replicates, and five repeats per line in each replicate, leading to 32 hydroponic trays, each accommodating up to 100 seedlings. To compare the differences among the lines, the relative chlorophyll concentrations were measured non-destructively from the base, middle, and tip of the uppermost leaves of each plant, and the average values were expressed as SPAD units (SPAD-502 chlorophyll meter, Minolta Camera Co., Ltd., Japan) as the indicator of leaf senescence caused by toxic As treatment. The responses of the plants to the As treatments were evident at 18 days after treatment. Changes in the shoot and root length response to the As treatments were measured for each entry at 18 days after treatment. Shoot length was measured from the base of the plant to the tip of the longest leaf, whereas root length was measured from the base of the plant to the root tip. Three plants per entry per replicate were rinsed with deionized water to remove any excess nutrients sticking to the surface of the plants and then oven-dried for 3 days at 70°C to remove moisture. Before sample processing for As analysis, dry biomass was recorded.

### Field Screening for Arsenic Accumulation in Grain

Field screening for As accumulation in grain was conducted at the field station of IRRI during the 2014 dry season. IRRI is situated at latitude 14°13′N and longitude 121°15′E, and the paddy soil type is a Maahas clay loam, isohyperthermic mixed-type tropical soil with average total As content in soil ranging from 2.6 to 7.2 mg kg^−1^ of dry soil, and irrigation water As concentration ranging from 0.021 to 0.047 mg L^−1^ (data not shown). Twenty-one-day-old seedlings were transplanted to the field with the experiment laid out in a randomized complete block design in two-row plots with 12 plants per row at a spacing of 20 × 20 cm. Standard agronomic practices with optimum fertilizer application, irrigation, and plant protection measures were carried out to ensure a good crop growth cycle for grain development. At maturity, eight middle plants of each genotype were harvested in bulk. The harvested seeds were oven-dried at 50°C for 3 complete days and de-husked brown rice was analyzed for As accumulation.

### Analytical Procedure for Determining Arsenic Content

The root and shoot samples from the hydroponic screening experiment and grain samples from the field experiment were analyzed for total As content. Dried samples were thoroughly homogenized by an ultra-centrifuge mill (ZM100, Retsch, Haan, Germany) modified with a tungsten blade to avoid any cross-contamination. Ground 0.2–0.5 g of sample was added to a closed-vessel digester, with 5 ml of high-purity 69% concentrated nitric acid (HNO_3_), followed by 2 ml of hydrogen peroxide (H_2_O_2_), and 1 ml of deionized water was added and pre-digested overnight in the fume hood (Amaral et al., 2013). On the following day, the samples were digested using a heating block at 150–155°C for 3 h under the fume hood. The digested tissue was diluted to a final volume of 25 ml using deionized water and total As was determined using Graphite Furnace Atomic Absorption Spectrophotometry (GFAAS-7000F, Shimadzu Corporation, Kyoto, Japan) (Hirohashi et al., 2001). Deuterium lamp background correction with high-performance As-hollow cathode lamps (193.7 nm) was used to quantify the As content in the known standards and unknown samples. An aliquot of 20 μl of the digested sample and 10 μl of palladium (100 ppm) as matrix modifiers were injected into the Pyrol-coated graphite tube with the aid of an autosampler unit. A seven-stage furnace cycle program was adopted with an atomization temperature of 2,200°C at the sixth stage and was used as a standard parameter to determine As content in the sample and the final concentration was expressed in milligrams per kilogram (mg kg^−1^).

### Statistical Analysis

For the germination screening experiment, three true treatment replicates were placed randomly in the plant growth chamber, with each replicate containing all 53 genotypes in an independent Petri dish holding 50 healthy seeds. For seedling-stage screening, the experiment was laid out as a two-factorial (genotype and treatment) design with three true treatment replicates (independent containers) and three randomized blocks within each treatment set containing all 53 genotypes. Two-way ANOVA was carried out to observe the effects of lines, treatment, and line-treatment interaction on different As stress-induced traits, and Tukey's honestly significant difference test was used for *post hoc* comparison of means (Table 2). For grain As content from the field experiment, three centered plants were analyzed separately for As content in grain, and Student's *t*-test was carried out to observe the variation in As accumulation between genotypes. All the statistical procedures were carried out using PBTools package of R-program (R Core Team, 2015). Pair-wise Pearson's correlation coefficient analysis was carried out among the As-related traits, in which *P-*value was two-tailed with two significant levels using *p* = 0.05 and *p* = 0.01, and a heat map was generated using *corrplot* package in R studio. Principal component analysis (PCA) was carried out to observe the pattern of variation among the 53 rice genotypes, the relationship among individuals, and their characteristics. Originally, relative index values were derived by assessing the response to the control value for respectively measured traits (germination capacity, chlorophyll content, plant height, and biomass) and absolute values for As content in shoot, root, and grain. Then, the index and absolute values from different screening strategies were combined to identify the correlation of the response variable vectors and genotypes. The analyses were performed using JMP^®^, Version <*16*> (SAS Institute Inc, 2014, Cary, NC, 1989–2019), and the results were summarized in biplots.

**Table 2:** Descriptive statistics and ANOVA results for measured phenotypes.

**Trait**		**Germination (%)**	**Chlorophyll content (SPAD)**	**Plant height (cm)**	**Root length (cm)**	**Shoot dry weight (g)**	**Root dry weight (g)**	**Arsenic content of shoots (mg kg^**−1**^)**	**Arsenic content of roots (mg kg^**−1**^)**
Control	Min	30	29.43	20.36	9.50	1.91	0.94	ND	ND
	Max	100	39.91	68.23	18.83	2.80	1.43	ND	ND
	Mean	85.11	35.31	44.75	14.42	2.35	1.19	ND	ND
Treatment 5 ppm As	Min	10	26.30	25.67	10.17	0.92	0.85	9.25	98.25
	Max	92	39.84	58.72	17.83	2.39	1.44	19.84	123.83
	Mean	60.69	33.26	41.87	13.49	1.60	1.12	14.79	111.4
Treatment 10 ppm As	Min	0	23.31	26.00	9.83	0.71	0.40	12.37	115.77
	Max	76	37.91	59.76	16.83	1.78	0.56	22.95	137.65
	Mean	41.19	31.75	43.34	13.31	1.23	0.48	18.05	126.69
Treatment 15 ppm As	Min	0	9.18	13.83	8.00	0.65	0.34	16.08	119.86
	Max	69	32.78	42.49	15.67	0.96	0.52	27.85	146.54
	Mean	27.3	21.69	28.03	11.10	0.80	0.43	22.46	133.77
Treatment 20 ppm As	Min	0	NA	NA	NA	NA	NA	NA	NA
	Max	59	NA	NA	NA	NA	NA	NA	NA
	Mean	16.06	NA	NA	NA	NA	NA	NA	NA
ANOVA result	G	***	***	***	***	***	***	***	***
	T	***	***	***	***	***	***	NA	NA
	G*T	***	***	***	***	***	***	NA	NA

Significance levels are indicated at p < 0.001/^***^ Treatment and genotype by treatment interaction effects were not analyzed for carcinogenic arsenic (As) content traits since in control conditions arsenic is not measured. ND, not determined; NA, not applicable; G, genotype; T, treatment; SPAD, soil plant analysis development; mg kg^−1^, milligrams per kilogram; cm, centimeter; g, gram; As, arsenic; ppm, parts per million; and ANOVA, analysis of variance.

## Results

### Effect of Arsenic on Seed Germination

**Figure 1:**
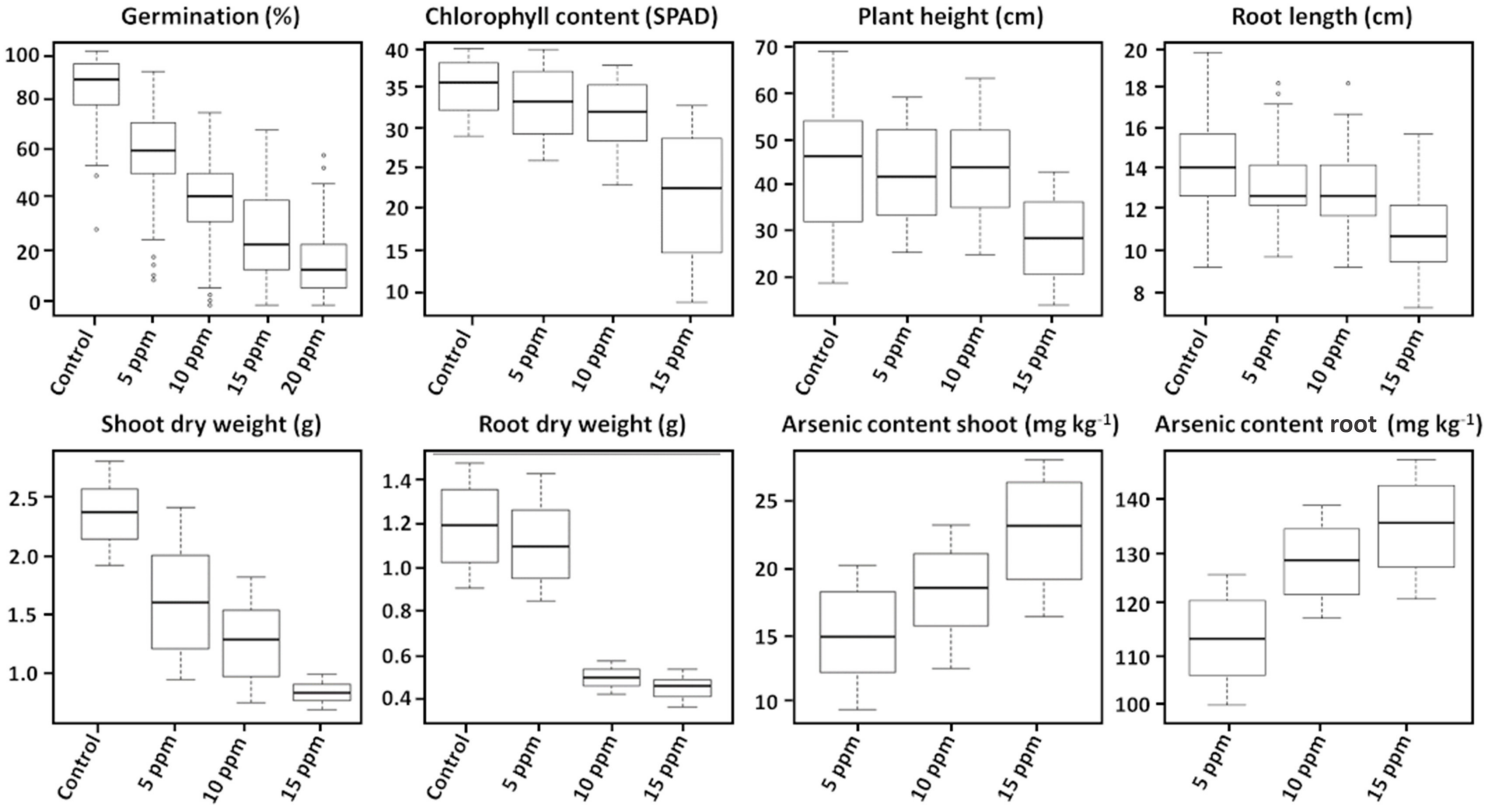
Box plot showing the distribution of phenotype values in 53 different cultivars under arsenic stress. The thick line in the middle of the box is the median of the distribution while the lower and upper boundaries represent the first and third quartile, respectively. Lower and upper whiskers are calculated based on 1.5 times the inter-quartile range. Control, without arsenic treatment; 5, 10, 15, and 20 ppm represent different concentrations of arsenic treatments (*n* = 3).

**Figure 2:**
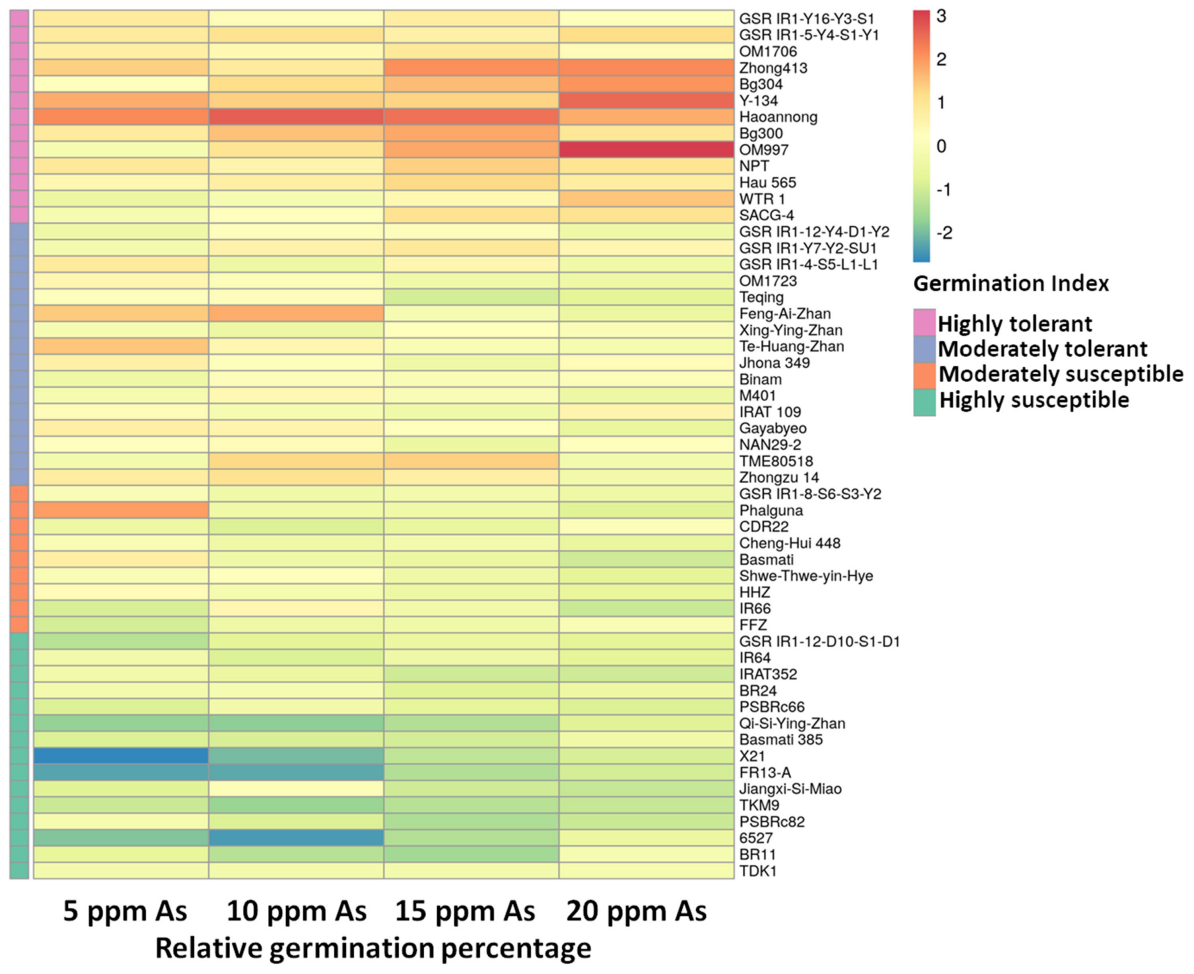
Grouping of 53 different cultivars based on relative germination tolerance under different arsenic stress. As, arsenic; ppm, parts per million.

Varying As stress induced substantial disruption in germination ability among the studied 53 genotypes. The germination percentages of different rice genotypes had different responses to As stress and germination decreased significantly with increasing As concentration (*p* < 0.01) (Figure 1). The mean (average value of 53 genotypes) germination ability over the control declined significantly with increasing As concentration in the treatment 0 ppm As (85.11%) **<** 5 ppm As (60.69%) **<** 10 ppm As (41.19%) **<** 15 ppm As (27.30%) **<** 20 ppm As (16.06%). The 53 genotypes were grouped into four clusters based on the relative tolerance percentage under varying As treatments (Figure 2). Genotypes showing a high tolerance index of >80% to low-As stress were clustered into highly tolerant genotypes. Genotypes exhibiting a poor tolerance index of <50% (lethal dose 50) in low-As stress treatment were classified as highly susceptible genotypes. Genotypes exhibiting tolerance only in lower concentration As treatments (5 and 10 ppm) were classified as moderately tolerant and genotypes exhibiting susceptibility from 10 ppm As treatment onward as moderately susceptible. Relative germination percentage among genotypes in the 5 ppm As treatment ranged from 29.5% in genotype X21 to 104.5% in Haoannong. In the 10 ppm As treatment, genotype Kelmekri 77/5 recorded the most decreased tolerance of <1% germination and genotype Haoannong showed high tolerance of 101%. In the 15 ppm As treatment, *indica* variety BR11 from Bangladesh failed to germinate even after 10 days of incubation period. However, *japonica* genotype Haoannong showed exceptional tolerance of 82.5% in the 15 ppm As treatment. In the 20 ppm As treatment, most of the evaluated genotypes had a tolerance percentage below 20%. However, *indica* genotype OM997 from Vietnam showed significant tolerance of 68.09% in 20 ppm As stress (Supplementary Data 1). Except for genotype Haoannong, all the genotypes from the *japonica* subspecies of cultivated rice *Oryza sativa* were highly susceptible under very high As stress. High-yielding mega-variety IR64 was moderately susceptible for germination under As toxicity stress. Genotypes Bg300 and Bg304 from Sri Lanka showed a higher tolerance under varying As stress. Bangladeshi genotypes BR11 and BR22 were highly susceptible for germination under varying As stress. Among the studied seven GSR-bred varieties, GSR12 exhibited high susceptibility and GSR8 was moderately susceptible to germination under varying As stress. However, GSR IR1-17-Y16-Y3-S1 and GSR IR1-5-Y4-S1-Y1 were highly tolerant across the varying As stress, and GSR IR1-12-Y4-D1-Y2, GSR IR1-5-Y7-Y2-SU1, and GSR IR1-4-S5-L1-L1 were moderately tolerant of As stress.

### Effect of Arsenic on Seedling Growth Parameters

**Figure 3:**
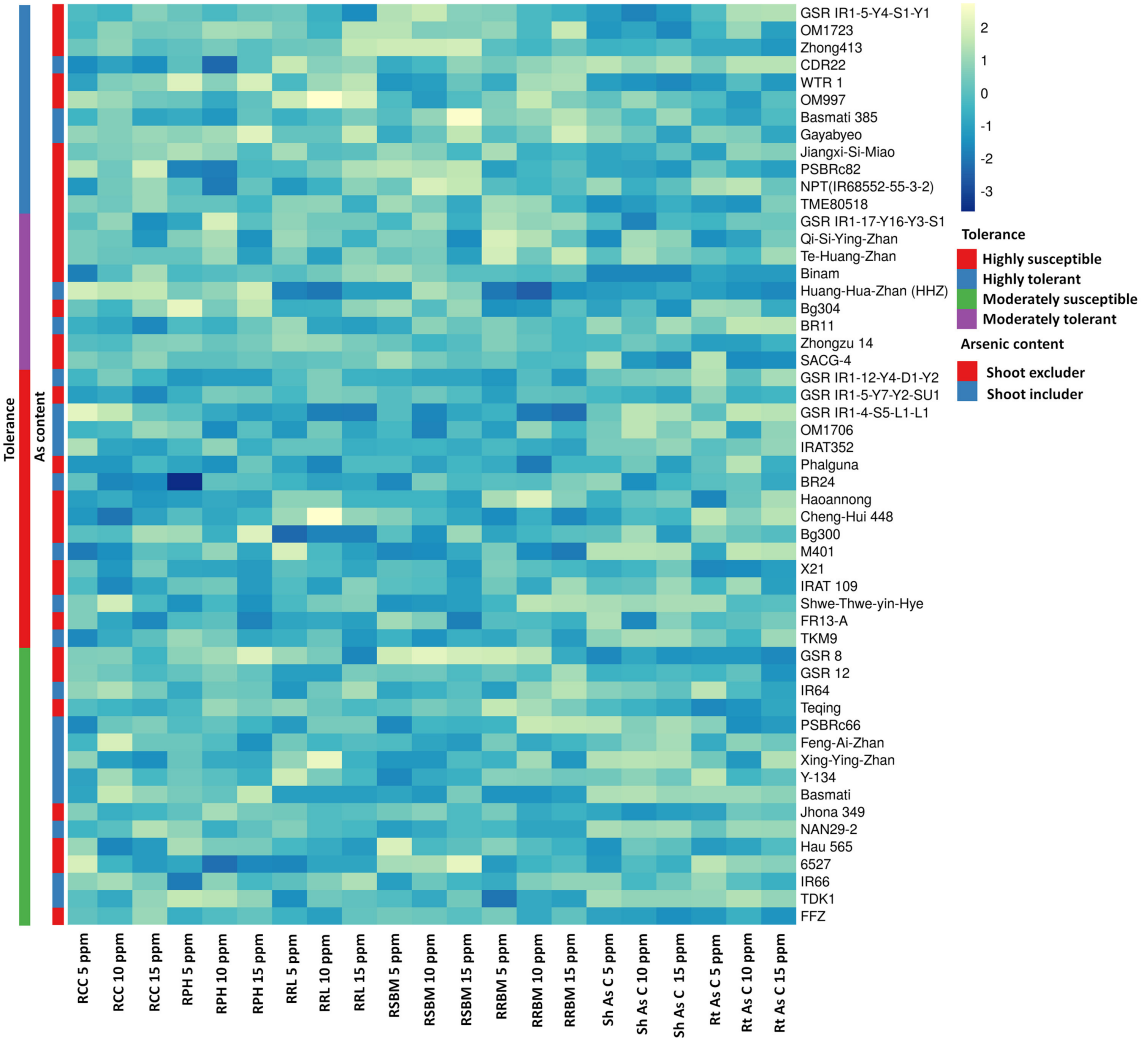
Grouping of 53 cultivars of rice (*Oryza sativa* L.) for traits associated with carcinogenic arsenic (As) toxicity stress for 18 days. As, arsenic; PPM, parts per million; RCC, relative chlorophyll content; RPH, relative plant height; RRL, relative root length; RSBM, relative shoot biomass; RRBM, relative root biomass; ShAsC, As content in shoots; RtAsC, As content in roots.

Exposure to varying concentrations of As for 18 days induced a significantly negative response among the investigated 53 genotypes. Chlorophyll content, plant height, and root length showed significant variation between the control and 15 ppm As treatment. Root and shoot length and biomass accumulation were negatively influenced by As treatment. Accumulation of As in the shoot and root tissue increased significantly with the increase in As in the treatment (Figure 1). There were significant genotypic differences in As accumulation in young seedlings after 18 days of As treatment. Based on the relative phenotypic performance of plants under varying As stress, genotypes were clustered into four different groups. Genotypes showing high tolerance (>80%) of As stress were grouped into highly tolerant genotypes, and genotypes exhibiting poor tolerance percentage (<50%; lethal dose 50) across all the As stress treatments were grouped into highly susceptible genotypes. Genotypes only exhibiting tolerance in lower concentration As treatments (5 and 10 ppm) were classified as moderately tolerant genotypes, and genotypes exhibiting susceptibility from 10 ppm As treatment onward as moderately susceptible genotypes. Further, genotypes were classified into two groups based on As accumulation in the shoots of the genotypes after 18 days of treatment: As shoot includers (>12 mg kg^−1^), and As shoot excluders (<12 mg kg^−1^) (Supplementary Data 2). The 53 genotypes were grouped into four clusters based on the relative tolerance percentage and shoot As accumulation under varying As treatments (Figure 3). In the 5 ppm As treatment, shoot As content ranged from 9.25 mg kg^−1^ in Binam to 19.84 mg kg^−1^ in CDR22, and root As content ranged from 98.25 mg kg^−1^ in Haoannong to 123.83 mg kg^−1^ in Cheng-Hui 448. In the 10 ppm As treatment, shoot As content ranged from 12.37 mg kg^−1^ in GSR IR1-5-Y4-S1-Y1 to 22.95 mg kg^−1^ in OM1706, and root As content from 115.77 mg kg^−1^ in X21 to 137.65 in BR11. In the 15 ppm As treatment, shoot As content ranged from 16.08 mg kg^−1^ (Binam) to 27.85 mg kg^−1^ (Xing-Ying-Zhan) and root As content ranged from 119.86 mg kg^−1^ (Huang-Hua-Zhan) to 146.54 mg kg^−1^ (BR11). The overall results indicated that adequate genetic variability is present among the studied genotypes for As accumulation in young seedlings for As stress. The concentrations of As in the seedlings of the 53 genotypes are presented in Supplementary Data 2. Genotypes displaying high susceptibility (M401 and TKM9) are As shoot includers and tolerant genotypes tended toward restricting As uptake in the shoot (NPT-IR68552-55-3-2, PSBRc82, and OM997). Genotypes Haoannong, BG300, and Phalguna showed higher susceptibility despite excluding As in the shoot. Genotype Basmati 385 (Aromatic subspecies) from Pakistan was highly tolerant despite including As in the shoot system (Figure 3). Genotypes NPT-IR68552-55-3-2, OM997, Zhong413, and WTR1 (BRRI dhan69) were highly tolerant by excluding As in the shoot system.

### Genotype Classification Based on Grain Arsenic Content

**Figure 4:**
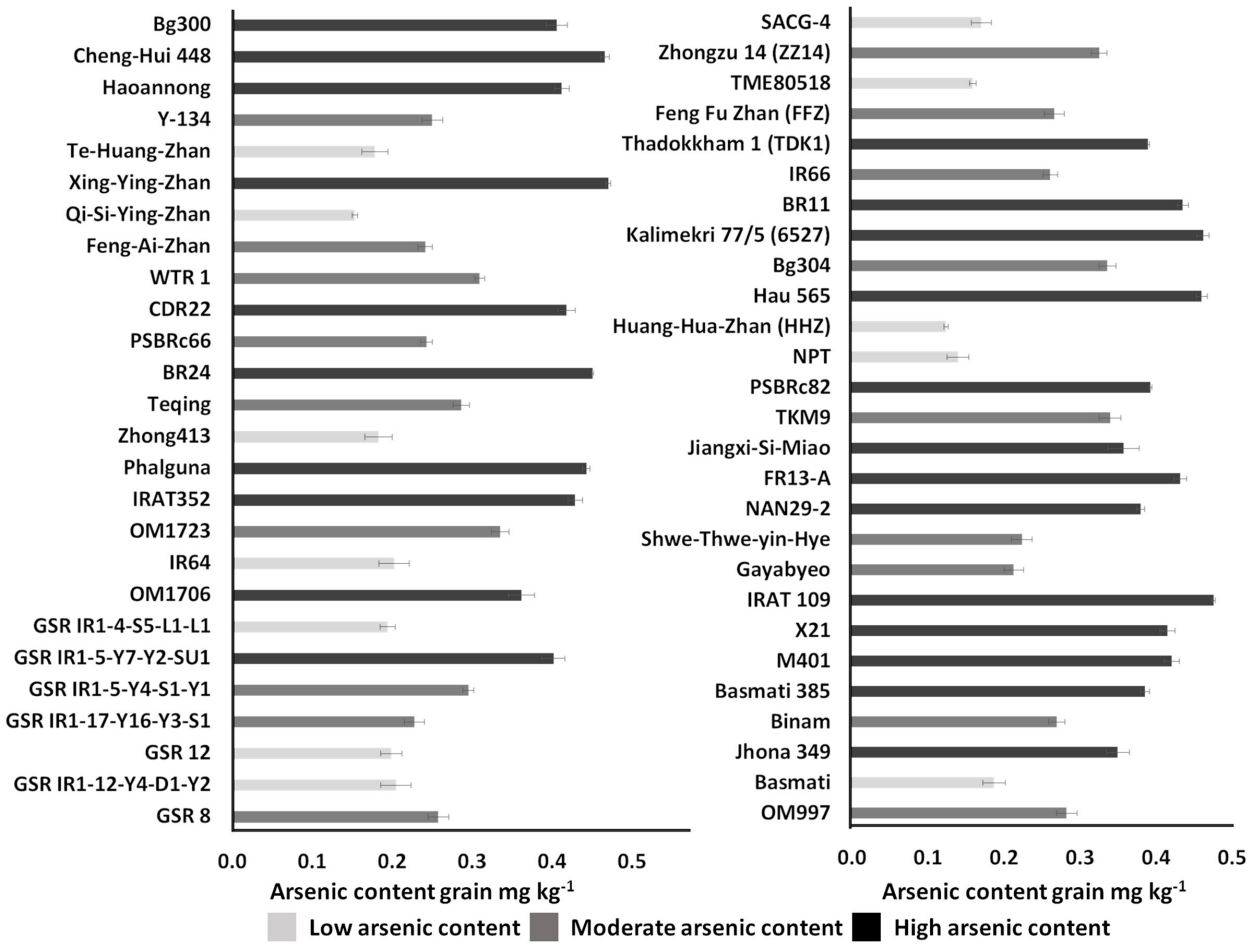
Classification of diverse rice genotypes based on arsenic content in unpolished brown rice. Error bar in the graph represents a standard error (*n* = 3); mg kg^−1^, milligrams per kilogram.

Unpolished brown rice of the 53 genotypes harvested from the field with soil As content <7 mg kg^−1^ also showed higher variation among the genotypes in grain As accumulation (Figure 4). Among the tested 53 genotypes, As content in grain ranged from 0.12 mg kg^−1^ in Huang-Hua-Zhan (*indica* subspecies) from China to 0.48 mg kg^−1^ in IRAT 109 (*Tropical japonica* subspecies) from Brazil. Based on As accumulation in the grain, the genotypes were classified into three groups: low As content rice (<0.2 mg kg^−1^), moderate As content rice (<0.3 mg kg^−1^), and high As content rice (>0.3 mg kg^−1^) (Figure 4; Supplementary Data 3). The rice genotypes of Bangladeshi origin, BR11 and BR24, accumulated >0.4 mg kg^−1^ of As in the grain. Genotypes Jhona 349, NAN29-2, Basmati 385, and Bg300 tended to accumulate moderate As in the grain (>0.2 mg kg^−1^). Genotypes from *indica* subspecies (Huang-Hua-Zhan, IR64, GSR IR1-12-Y4-D1-Y2, GSR 12, GSR IR1-4-S5-L1-L1, SACG-4, TME80518, Qi-Si-Ying-Zhan, Te-Huang-Zhan, Zhong413, and Basmati) and *japonica* subspecies (NPT-IR68552-55-3-2) accumulate less As (<0.2 mg kg^−1^) in the grain. High-yielding mega-variety IR64 also accumulated less As (<0.2 mg kg^−1^) in the grain.

### Correlation Among Measured Traits

Pearson's *r* correlation coefficient among various measured As stress-responsive traits from germination tolerance in the early growth stage of rice, and grain As content screening revealed the existence of a complex association between the studied traits (Supplementary Figure 1). Significant negative correlations were observed between As content in grain from field screening and relative chlorophyll content (10 and 15 ppm As) at the early growth stage of rice. Also, there exists a significant negative correlation between As content traits and other measured morphological parameters at the early growth stage of rice. However, a significant positive correlation exists between root As content and shoot As accumulation. Significant positive correlation of decreasing order (0.78 > 0.63 > 0.45) was observed between 5 ppm relative germination index and 10, 15, and 20 ppm relative germination index. No significant correlation was observed between relative germination indices and any of the other measured traits.

### Genotype Assessment Using Principal Component Analysis

**Figure 5:**
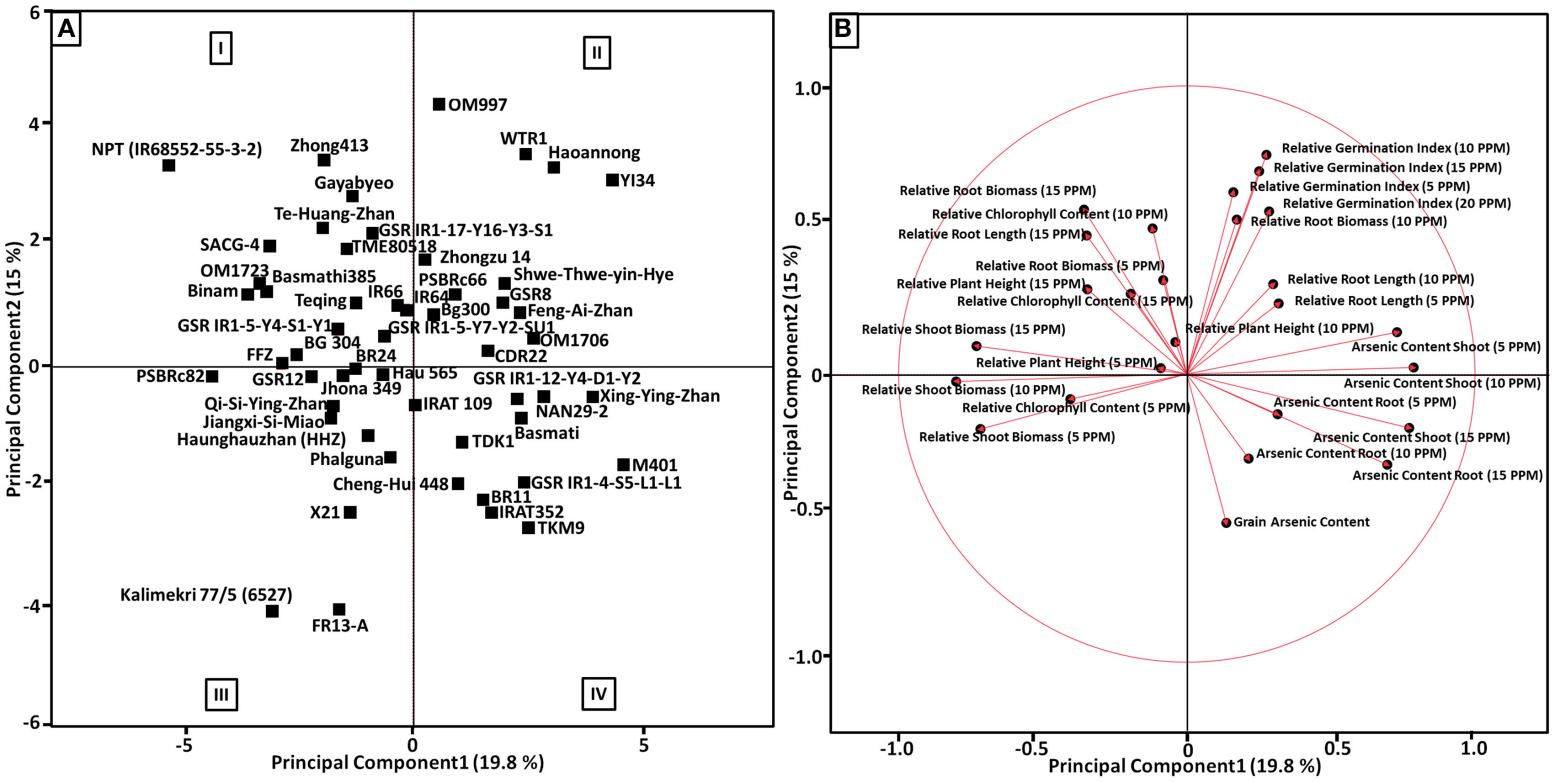
**(A)** Score plot showing principal component analysis (PCA) for the first two principal component (PC) scores, PCA1 vs. PCA2 describing the morpho-physiological parameters measured for all 53 genotypes under germination tolerance, the early growth stage of rice and As accumulation characteristics. Rice genotypes with high/low scores in each tolerant category are identified in the PCA plots. (I) Genotypes with strong early development stage tolerance, moderate to high germination tolerance, and accumulating less As in root, shoot and grain. (II) Genotypes with strong germination stage tolerance, high to moderate tolerance in early development and accumulate less to moderate As in root, shoot and grain. (III) Genotypes having moderate to low tolerance in the early stages of development, and high sensitivity toward germination tolerance, and accumulating moderate to high As in root, shoot and grain. (IV) Genotypes having moderate to high sensitivity toward germination tolerance, high sensitivity toward the early stages of development, and accumulating high As in root, shoot and grain. **(B)** Loading plot showing the vector coefficients of As-related trait variables for the first principal component vs. the coefficient for the second principal component.

Principal component analysis was performed to identify the principal components of As content traits (grain, shoot, and root), relative germination indices (5, 10, 15, and 20 ppm As) and relative root-shoot morphological parameters (5, 10, and 15 ppm As) of 53 rice genotypes, and PCA best describes the response to identify As-tolerant and low-accumulating genotypes. Of the total variation (34.8%), the first two principal components (PCs) accounted for 19.8 and 15% among the genotypes, respectively (Figure 5). The first PC represents a higher value for As content in shoot traits (10, 15, and 5 ppm), As content in root traits (15 and 5 ppm), and relative root length 10 ppm, but lesser loading values for relative germination index (5, 10, 15, and 20 ppm), As content in root 10 ppm, relative biomass 10 ppm, and grain As content. The second P showed higher loading values for relative germination index (10, 15, 20, and 5 ppm), relative root biomass (15, 10, and 5 ppm), relative chlorophyll content 10 ppm, and relative root length 10 ppm, but lesser loading values for relative plant height (15, 10, and 5 ppm), relative chlorophyll content 15 ppm, relative root length 5 ppm, shoot As content (5 and 10 ppm), and relative shoot biomass 15 ppm (Figure 5B). A biplot of PC1 vs. PC2 divided the 53 genotypes into different As-tolerant and As-accumulator groups (Figure 5A). A total of 34.8% of the variation was collectively explained by PC1 and PC2 and helped to classify genotypes according to tolerance level and As accumulation. Genotypes with a high tolerance for early stages of development, moderate to high germination tolerance, and low As accumulation in the root, shoot, and grain, which appear in the upper-left quadrant of the PCA graph, were regarded as the most suitable for breeding in the As-contaminated rice growing ecosystem. Genotypes having moderate to high sensitivity toward germination tolerance, high sensitivity toward the early stages of development, and accumulating high As in root, shoot, and grain, which appear in the lower-right quadrant of the PCA graph, were regarded as the most sensitive genotypes accumulating high amount of As. Genotypes with moderate to high sensitivity to germination tolerance, high sensitivity to early stages of development, and high As accumulation in the root, shoot, and grain, which show up in the lower-right quadrant of the PCA graph, were considered the most sensitive genotypes accumulating high As. The top right portion of the PCA graph showed genotypes with high germination stage tolerance, high to moderate tolerance in early development, and low to moderate accumulation in the root, shoot, and grain. Genotypes with moderate to low tolerance in the early phases of development, high sensitivity to germination tolerance, and moderate to high As accumulation in the root, shoot, and grain, are placed in the bottom-left quadrant of the PCA graph (Figure 5A).

## Discussion

In this current study, we adopted three different types of screening strategies to identify germplasm suitable for growing in As-contaminated ecosystems. Germination screening was conducted in a plant growth chamber, where seeds were exposed to varying As concentration to identify germplasm that tolerates As and shows optimal germination capability (Hossain et al., 2007). Because of the labor shortage, rice production is shifting toward the most cost and water-efficient DSR methods (Mishra et al., 2017). Therefore, it is important to identify germplasm that can tolerate excess As present in soil and establish seeds in As-contaminated ecosystems (Murugaiyan et al., 2019). For the seedling-stage screening experiment, a hydroponics system was adapted in a controlled phytotron glasshouse to eliminate the interaction of other environmental factors (Amaravel et al., 2019). Seven-day-old seedlings were exposed to varying As concentrations for 18 days to identify germplasm that tolerates a high amount of As by excluding it in the shoot. This experiment suggests that most of the As load is retained in the root system and a very small portion is available for translocating in the shoot system (Tuli et al., 2010; Suriyagoda et al., 2018). For As accumulation in grain, a field screening experiment with optimum rice-growing and irrigation practices was adopted to identify germplasm that accumulates less As in grain. Inorganic arsenic species tend to dominate the major rice-growing ecosystems. In flooded paddy soils, arsenite^(III)^, a neutral molecule is the most dominating As species. It enters plants through aquaporin channels, primarily through the nodulin 26-like intrinsic proteins (NIPs, a subfamily of the aquaporin family) (Carey et al., 2010). Arsenate^(V)^ is the most prevalent As species in aerobic soils, accounting for just a small proportion of total As in flooded paddy soils. However, in rice, *OsHAC1;1* (*Loc_Os02g01220*) and *OsHAC1;2* (*Loc_Os04g17660*) are responsible for As^(V)^ reduction to As^(III)^ to facilitate As^(III)^ efflux out into the external environment (Shi et al., 2016). Therefore, sodium-meta-arsenite (AsNaO_2_) was used in the treatment in this study (Murugaiyan et al., 2019). Most natural soils contain low concentrations of As. Background concentrations in agricultural soil range from 1 to 40 mg kg^−1^, with a mean value of 5 mg kg^−1^, and As accumulation in grain is directly proportional to the As bioavailability in the soil (Abernathy et al., 2001; Wong et al., 2012).

Germination assays are not a commonly used technique for testing As toxicity in rice. However, in As-contaminated soils, seed germination capability is one of the best indicators for the successful or unsuccessful establishment of a plant (Abedin et al., 2002a). In this study, we exposed 53 genotypes to varying concentrations (5, 10, 15, and 20 ppm) of As^(III)^ at the germination stage for 10 days. Similar concentrations frequently occur in the topsoil of the rice-growing regions of Bangladesh and West Bengal, India. The presented results revealed the toxic effect of As and germination ability decreased significantly with increasing As concentration in the treatment [0 <5 <10 <15 <20 ppm As^(III)^] which is consistent with the results of previous studies (Abedin et al., 2002b; Halim et al., 2016). Arsenic is known to inhibit the overall germination ability of rice plants by acting as a metabolic inhibitor (Azizur Rahman et al., 2007; Shri et al., 2009). In our study, we found that the treatment with 10 ppm had 50% lethal effects on the germination of seeds in most of the rice genotypes. In the 15 and 20 ppm As treatments, the germination capacity of much germplasm fell below 20% and these results confirm the toxic effect of As on seed germination and correlate with a previous study (Hossain et al., 2007). However, Hossain et al. (2007) reported that germination failed completely in the 10 ppm As treatment, whereas, in our study, some germination at that concentration was still observed. In the same study involving BR11 germplasm, Hossain et al. (2007) reported that germination failed in the 10 ppm As treatment, but in our study, BR11 showed 48% germination in the 10 ppm As treatment and then failed to germinate in the 15 ppm As treatment.

In hydroponics As stress, irrespective of the rice genotype, the overall plant performance decreased with an increasing concentration of As in the nutrient solution (Figure 2). Arsenic accumulation was relatively higher in the root than in the shoot (root > shoot), in agreement with the results of prior studies (Raab et al., 2007; Lu et al., 2010). However, considerable genotypic variation was observed among the studied genotypes for As tolerance and accumulation in the shoot (Figure 3; Supplementary Data 2). In general, from the hydroponic screening experiment, it was noted that genotypes including As in the shoot displayed decreased As tolerance. In a prior study, it was observed that mega-variety IR64 was susceptible to As^(V)^ treatment with high accumulation in the shoot (Tripathi et al., 2012). In our experiment, IR64 displayed tolerance toward As despite including it in the shoot, in agreement with the results of a previous study (Dasgupta et al., 2004). Surprisingly, IR64 was one of the genotypes with the lowest As in the unpolished grain when grown in the field (<0.20 mg kg^−1^).

Genotypic differences in grain As accumulation by rice genotypes have been reported by several field studies (Kuramata et al., 2011; Islam et al., 2016; Liu et al., 2019; Fernández-Baca et al., 2021). Also, it has been reported that *indica* cultivars tend to accumulate higher amounts of inorganic As in grain and shoot than *japonica* cultivars (Suriyagoda et al., 2018). However, most of the reported *japonica* cultivars were not adapted to grow in the highly contaminated tropical and subtropical flood plains of Asia. In our study, rice germplasm was cautiously selected to integrate into the ongoing breeding programs at IRRI targeting As-contaminated regions. Total As concentration in the unpolished grain from the 53 genotypes (Figure 4) (brown rice) ranged from 0.12 to 0.48 mg kg^−1^ with an average value of 0.31 mg kg^−1^. Most of the studied genotypes surpass the allowed maximum level of inorganic As of 0.20 mg kg^−1^ set by the Codex Alimentarius Commission-United Nations food safety standards (Pillai et al., 2010; Stankovic, 2016; Islam et al., 2017). It is also evident from our experiment that the tested genotypes (BR11 and BR28) from Bangladesh accumulate more As in the grain (>0.40 mg kg^−1^) and displayed strong susceptibility in germination and seedling-stage experiments as they were unable to exclude As from the shoot system. Similar results were observed with the same genotypes in field-based experiments (Azizur Rahman et al., 2007; Ahmed et al., 2011). Most of the *japonica* subspecies genotypes tended to accumulate more As in the grain than the *indica* subspecies genotypes (Tuli et al., 2010). However, Qi-Si-Ying-Zhan, a *japonica* genotype from southern China, accumulated less As (<0.12 mg kg^−1^) in grain and also displayed moderately resistance in hydroponic screening by excluding As in the shoot, but it displayed susceptibility in germination tolerance. In our study, we explored the genotypes commonly used by breeders to develop varieties for Bangladesh and India. In developing rice-growing Asian countries, straw is either burned in the field or used as fodder for cattle, which further increases the risk of As exposure (Njie and Reed, 1995; Lawler and Dritz, 2005).

Pearson's *r* correlation coefficient among various measured As stress-responsive traits from germination tolerance, the early growth stage of rice, and grain As content screening revealed the existence of a complex association between the measured traits (Supplementary Figure 1). This is of great importance for foreseeing the association between multifaceted quantitative As content traits and simple quantitative morphological traits associated with As stress (Murugaiyan, 2019; Murugaiyan et al., 2019). Our study revealed a significant interrelationship among various As accumulation and morphological components. The significant negative correlation between As content traits and relative plant morphological traits signifies that As content in plant parts is inversely proportional to the relative morphological traits. An increase in relative chlorophyll content significantly increases biomass accumulation and decreases As content in shoot and grain, suggesting that most genotypes that exclude As in the system were tolerant. These results were confirming the findings of many previous experiments conducted to identify toxic effects of As, in which As is known to interact deleteriously with rice plants by decreasing chlorophyll content and biomass (Panaullah et al., 2009; Das et al., 2013). From our study, it can be established that genotypes exhibiting tolerance by excluding As in the shoot at the seedling stage also accumulated less As in the grain. Correspondingly, As content in shoot and root was positively correlated with increasing As concentration in the treatment, signifying that increased As content will be quite damaging to rice plant health. From our experiment, it is evident that relative germination index traits were independent of the traits from the early growth stage of rice and grain As content. However, a weak negative correlation exists between germination index at 15 ppm and relative shoot biomass at 10 ppm. Previously, principal component analysis has been used effectively in rice to categorize genotypes into different drought, salinity, and disease tolerance groups (Li et al., 2018; Kakar et al., 2019). By applying this multidimensional preference analysis, we try to identify parameters that are best described using As tolerance and accumulation for response variables (Figure 5). Toxic symptoms associated with As stress such as plant growth reduction, decreased biomass, and impairment of the photosynthetic process have been reported widely in rice (Abedin et al., 2002a; Meharg and HartleyWhitaker, 2002; Azizur Rahman et al., 2007; Murugaiyan et al., 2019). However, screening strategies to identify the most preferable genotypes for As-contaminated ecosystems were not appropriately established. In our study, PCA and correlation analysis indicated that As content traits from the shoot and root were clustered together, suggesting that they are significantly connected, even more so than grain As content, morphological, and relative germination parameters. Furthermore, all of the relative germination characteristics were grouped together, implying that relative germination parameters were unaffected by As content or morphological factors. We also found that relative chlorophyll content parameters are loaded in the PCA plot against the As content traits, suggesting that chlorophyll content traits are more important in identifying tolerant and low-As-accumulating rice genotypes. These results were confirming the findings of many previous experiments that stunted growth and decreased photosynthesis are the major toxicity symptoms of As interaction with rice (Dasgupta et al., 2004; Abbas et al., 2018; Norton et al., 2019).

Carcinogenic arsenic accumulation in rice plants damages tissue and disrupts cellular homeostasis. As toxicity in rice plants has a cascade effect that includes preventing the most sensitive germination phase, limiting cell division, elongation of the primary roots, stunted development, and, finally, grain accumulation (Suriyagoda et al., 2018; Murugaiyan et al., 2021). As absorption by the roots and transport to the leaves via the transpiration stream via xylem resulting in a toxic As load in plant tissues (Tang et al., 2020). The presence of arsenic toxicity characteristics in rice germplasm under our experimental circumstances indicates that As tolerance and accumulation in rice is a quantitatively inherited trait influenced by multiple factors. Overall, choosing genotypes with germination tolerance, As excess tolerance by As exclusion in the root, or As excess tolerance by As retention in the root and avoidance of As translocation to shoot will minimize carcinogenic As load in the edible portions of the rice plant. Accumulation of As in the rice plant not only affects plant performance but also threatens the health of populations that depend on rice for their dietary supplies and livestock (Carbonell-Barrachina et al., 2015). Before nominating varieties for well-established As-contaminated ecosystems such as those of West Bengal, India, and Bangladesh, it might be beneficial to screen all genotypes against As at the germination stage, at the early seedling stage, and in field conditions to identify the most desirable genotypes.

## Conclusions

Our study demonstrates screening strategies for recognizing suitable tolerant and As-excluding germplasm, and offers great potential for use in targeted breeding programs for As-contaminated regions. Irrespective of the As concentration in the treatment, germination, overall plant health, and tolerance percentage declined drastically, signifying that the presence of As harms rice production. For future screening of rice germplasm, 10 mg kg^−1^ As treatment, slightly above the background concentration in the soil, seems to be the most suitable concentration for effective screening. Significant variation in As accumulation was observed in the grain. Low-accumulating genotypes can be used in breeding programs for identifying QTLs and genes responsible for As accumulation and translocation. Among all the materials studied, NPT (IR68552-55-3-2), WTR1, GSR IR1-5-Y4-S1-Y1, OM997, and Zhong413 were tolerant against a high concentration of As and they also accumulate very low concentrations of As in the grain. These identified As-tolerant genotypes would contribute greatly to the development of As-excluding varieties for contaminated ecosystems. Recently, genotype WTR1 was nominated in Bangladesh and released as BRRI dhan69, making a significant impact on mitigating the problem of As contamination.

## Data Availability Statement

The original contributions presented in the study are included in the article/Supplementary Material, further inquiries can be directed to the corresponding author.

## Author Contributions

JA, MF, and VM conceived the research. VM and JA created the rice diversity panel. VM conducted the As screening experiment and collected the data. VM, FZ, AW, AP, L-BW, and JM worked on the data analysis and drafting of the manuscript. VM, JA, MF, JM, and ZL reviewed the manuscript. All authors read and approved the manuscript.

## Funding

The authors would like to thank and acknowledge the Bill & Melinda Gates Foundation for providing a research grant to ZL for the Green Super Rice project under ID OPP1130530. The work reported in this manuscript was part of VM's Ph.D. thesis funded through the Green Super Rice project at IRRI (https://nbn-resolving.org/urn:nbn:de:hbz:5n-55609).

## Conflict of Interest

The authors declare that the research was conducted in the absence of any commercial or financial relationships that could be construed as a potential conflict of interest.

## Publisher's Note

All claims expressed in this article are solely those of the authors and do not necessarily represent those of their affiliated organizations, or those of the publisher, the editors and the reviewers. Any product that may be evaluated in this article, or claim that may be made by its manufacturer, is not guaranteed or endorsed by the publisher.

## Supplementary Material

Supplementary Figure 1 Heat map showing the Pearson correlation coefficient value of As-related traits. RCC, relative chlorophyll content; RPH, relative plant height; RRL, relative root length; RSBM, relative shoot biomass; RRBM, relative root biomass; ShAsC, As content in shoots; RtAsC, As content in roots; GrAsC, As content in grain; RGI, relative germination index. Correlation was significant at ^**^*p* < 0.01 and ^*^*p* < 0.05 and values in each rectangle indicate correlation coefficients.

Supplementary Data 1 Relative germination tolerance percentage of 53 genotypes under varying arsenic treatment (*n* = 3).

Supplementary Data 2 Mean relative phenotypic values of traits measured under 15 ppm arsenic stress (*n* = 3).

Supplementary Data 3 Mean arsenic content in brown rice harvested from field experiment (*n* = 3).
