# Supplementary figures and images for: Identification of Promising Genotypes Through Systematic Evaluation for Arsenic Tolerance and Exclusion in Rice (Oryza sativa L.)
Source: Front Plant Sci. 2021 Oct 29;12:753063. doi: 10.3389/fpls.2021.753063 (PMC8589031; doi:10.3389/fpls.2021.753063)

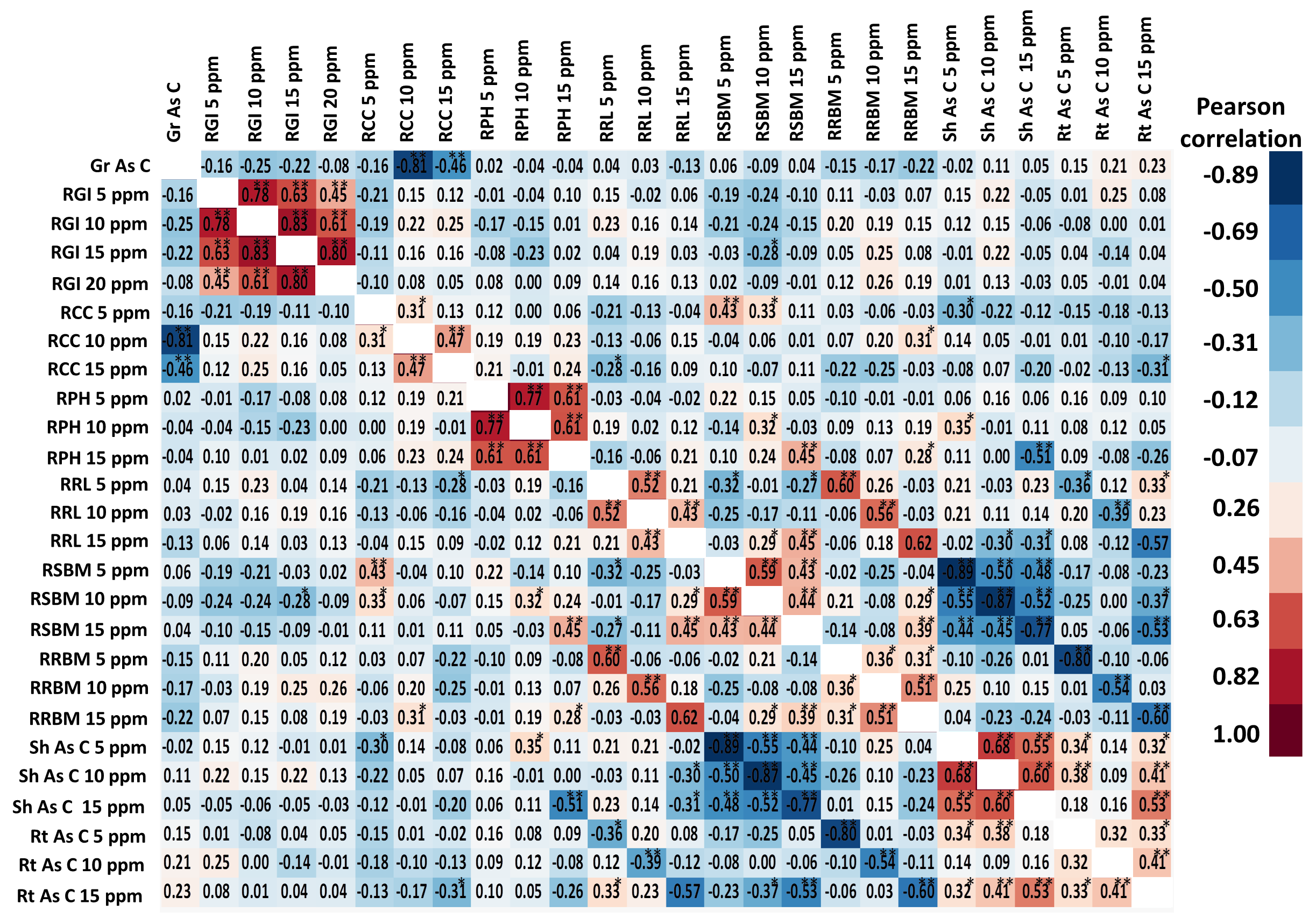

Supplement: Supplementary Figure 1 — Heat map showing the Pearson correlation coefficient value of As-related traits. RCC, relative chlorophyll content; RPH, relative plant height; RRL, relative root length; RSBM, relative shoot biomass; RRBM, relative root biomass; ShAsC, As content in shoots; RtAsC, As content in roots; GrAsC, As content in grain; RGI, relative germination index. Correlation was significant at **p < 0.01 and *p < 0.05 and values in each rectangle indicate correlation coefficients. [file Image_1.TIF]
